# The value of TDI combined with myocardial strain parameters in quantitative evaluation of left heart function in parturient with pregnancy-induced hypertension

**DOI:** 10.1038/s41598-023-48599-z

**Published:** 2023-12-03

**Authors:** Xiumei Lin, Chengwei Lu, Guifeng Ma

**Affiliations:** 1 Department of Ultrasound, The Affiliated Hospital of Weifang Medical University, 2428 Yuhe Road, Weifang, 261031 Shandong China; 2 Liutuan Town Central Health Center, Weifang, Shandong China

**Keywords:** Cardiology, Diseases, Medical research

## Abstract

This study aimed to investigate the value of tissue doppler imaging (TDI) and 4D myocardial strain parameters in evaluating left heart function of pregnant women with hypertension and the association between these parameters and relevant factors. Forty-five pregnant women with hypertensive disorder, including 20 with hypertension, 15 with mild preeclampsia, and 10 with severe preeclampsia, were recruited, and their cardiac functions were compared with those of 30 healthy pregnant women as controls. High Left ventricular end-systolic volume (LVESV), Left atrial volume index (LAVI), E/e were observed in hypertensive disorder, while Mitral peak diastolic velocity(E), Early diastolic peak velocity(e), E/A, Left ventricularglobal longitudinal strain (LVGLS), Left ventricularglobal area strain (LVGAS), and Left atrialglobal longitudinal strain (LAGLS) were decreased; for pre-eclampsia, Left ventricular end-systolic diameter (LVESD), Left atrial anteroposterior diameter (LAD-ap), LVESV, LAVI were significantly increased, LVGLS, LAGLS were significantly decreased, Left ventricular end-diastolic diameter (LVEDD), Left ventricular end-diastolic volume (LVEDV), A peak, E/e were increased, while E peak, E/A, e, Left ventricle global radial strain (LVGRS), Left ventricle global circumferential strain (LVGCS), LVGAS were decreased but not significantly; for severe preeclampsia, Left ventricular end diastolic diameter (LVEDD), LVESD, LAD-ap, Left ventricular end-diastolic volume (LVEDV), LVESV, LAVI, A, and E/e were significantly increased, while LVGLS, LVGRS, LVGCS, LVGAS, LAGLS, E peak, E/A, and e were significantly reduced. TDI combined with 4D myocardial strain parameters can detect early changes in cardiac function of hypertensive disorders in pregnancy, with LVGLS, LVGAS, and LAGLS being the most sensitive indicators for early changes. Such findings provide a basis for effective clinical treatment of these symptoms.

## Introduction

Hypertensive disorders in pregnancy (HDP) is a general term for a group of medical disorders clinically characterized into five diagnosable conditions, namely gestational hypertension, preeclampsia, eclampsia, chronic hypertension complicated by preeclampsia and combined chronic hypertension in pregnancy, with the two most frequently seen types being gestational hypertension and preeclampsia^1–3^. In normal pregnancies, the effective circulating blood volume increases, leading to an increase in the cardiac workload. If sustained hypertension persists during gestation, this could lead to a decrease in cardiac pump function. The heart and kidneys are known to interact and are interdependent organs whereby damage to either can lead to the other's functional abnormalities^4,5^. Conventional evaluation techniques such as electrocardiography (ECG) and two-dimensional echocardiography (2D-echo) are often used clinically to evaluate cardiac function; however, such methods are imprecise at detecting cardiac abnormalities at an early stage and thus may result in delaying diagnosis of the condition and subsequently resulting in irrevocable damage. To address this limitation, various ultrasound techniques have been introduced recently, most notably M-mode echocardiography, tissue Doppler imaging (TDI) technology, two-dimensional speckle tracking imaging (2D-STE), three-dimensional speckle tracking imaging (3D-STI), and the most recent method, four-dimensional speckle tracking imaging (4D-STI)^6–10^. Unlike conventional methods which assume left ventricular (LV) geometry, 4D-STI technology can rapidly track echogenic speckles in three-dimensional space and thus provide more precise and reproducible measurements of myocardial motion function and three-dimensional shape of the heart without any LV geometrical assumptions. Consequently, 4D-STI can help identify cardiac abnormalities at an early stage and facilitate a timely response to any clinical deterioration. In this study, we investigated the value of TDI and 4D myocardial strain parameters in evaluating left heart function of pregnant women with hypertension and the association between these parameters and relevant factors, in order to provide a basis for effective clinical treatment of these symptoms.

## Materials and methods

### General information

Forty-five patients, including 20 patients with gestational hypertension and 25 patients with preeclampsia, aged 20–35 years, mean age 26.60 ± 2.40 years, gestational weeks 20–40 weeks, mean gestational weeks 34.31 ± 3.20 weeks, were selected and diagnosed with hypertensive disorders in pregnancy in Our institute between March 2021 and October 2022. The criteria for diagnosing hypertensive disorders in pregnancy were in accordance with the diagnostic criteria of the eighth edition of the national undergraduate textbook of medical higher education, Obstetrics and Gynecology^11–14^. Ethical approvals were obtained from Ethics Committee of the Affiliated Hospital of Weifang Medical College (No.KY-2021-M14). Among them, the severity of preeclampsia was referred to the American College of Obstetricians and Gynecologists, which established the index of hypertensive disorders in pregnancy (2013 edition). In addition to meeting the above conditions, the following special conditions should be excluded: (1) primary hypertension, that is, hypertension that occurred before pregnancy or before 20 weeks of gestation; (2) pregnant women except for congenital heart disease, various types of cardiomyopathy, various valve diseases, various arrhythmias and other cardiac diseases; (3) pregnant women except for some endocrine diseases; (4) pregnant women except for some primary liver and kidney insufficiency; (5) Some special patients whose body size is too obese to be shown by transthoracic ultrasound. Inform the patient beforehand and obtain the patient’s informed consent.

Meanwhile, 30 healthy pregnant women, aged 20–35 years, mean age 27.33 ± 1.71 years, gestational weeks 20–40 weeks, mean gestational weeks 34.80 ± 2.94 weeks, were selected as the control group. The healthy control group underwent a series of cardiac examinations to ensure that no signs of hypertensive disorders in pregnancy were present throughout pregnancy.

### Apparatus and operation method

A GE Vivid E95 color Doppler diagnostic echocardiograph from GE, USA was used, with a 2D M5S-D cardiac probe and a 4D 4 V cardiac probe at 3.5 MHz, equipped with a 4D imaging software package and the offline quantitative analysis software Echo PAC. Two-dimensional echocardiography was performed at a frame rate of > 28 fps. LVEDD, LVESD, Ieft ventricular septal thickness (IVST), Left ventricular posterior wall thickness (PWT), Left ventricular ejection fraction (LVEF), Left atrial end-systolic anteroposterior diameter (LAD-ap), E, Late diastolic peak velocity (A), and E/A were measured. The TDI technique was used to measured and record e at the septum of the mitral annulus and E/e at the septum of the mitral annulus. Using a 4-dimensional 4 V cardiac probe, 4-dimensional imaging was performed after the subject's heart rhythm was stabilized. A two-dimensional standard four-chamber cardiac view is selected, with the entire myocardium included, and then the 4 V-D button is clicked to select 4–6 cardiac cycles at a frame rate greater than 40% of the heart rate, and the subject is asked to hold his or her breath for 10 s. The stored data is imported into Echo PAC software for offline analysis.

### Statistical methods

The data were analyzed using SPSS V22.0 statistical software, and the measurement data were shown as mean ± standard deviation. one-way ANOVA was used for comparison between multiple groups, independent sample *t* test was used for two-way comparison between groups, and Pearson method was used for correlation factor analysis. P < 0.05 was considered statistically significant difference.

### Ethics approval and consent to participate

Written informed consent was obtained from all patients recruited in this study. All methods were carried out in accordance with Declaration of Helsinki and Good Clinical Practice (GCP) guidelines. Institutional Review Board of the Affiliated Hospital of Weifang Medical University have approved the study protocol (Ethics Number: KY-2021-M14).

## Results

### Observation of parameters in these four groups of subjects under 2D conventional echocardiography

Compared with the control group, the E peak and E/A of pregnant women with gestational hypertension were lower than those of the control group, and the difference was statistically significant (P < 0.05); LVEDD, LVESD, IVST, PWT, LAD-ap, and A peak did not change significantly, and the difference was not statistically significant (P > 0.05); LVESD and LAD-ap of pregnant women with pre-eclampsia (mild) were significantly higher than those of the control group, and the difference was statistically significant (P < 0.01). LVESD and LAD-ap were significantly higher than those in the control group, and the difference was statistically significant (P < 0.01), LVEDD was higher than that in the control group, peak E and E/A were lower than those in the control group, peak A was higher than that in the control group, and the difference was statistically significant (P < 0.05), and IVST and PWT had slight changes compared with those in the control group, but the difference was not statistically significant (P > 0.05); LVEDD, LVESD, LAD-ap, and LAD-ap in pregnant women with preeclampsia (severe) were not statistically significant (P > 0.05), LVESD and LAD-ap were significantly higher than those in the control group, and the difference was statistically significant (P < 0.01), the E peak and E/A were significantly lower than those in the control group, the A peak was significantly higher than those in the control group, and the difference was statistically significant (P < 0.01), and the IVST and PWT were slightly increased than those in the control group, but the difference was not statistically significant (P > 0.05) (See Tables 1 and 2).

**Table 1:** Comparison of parameters under 2D conventional echocardiography in four groups of subjects (mean number ± standard deviation).

Projects	Number of cases (pcs)	LVEDD (mm)	LVESD (mm)	IVST (cm)	PWT (cm)
Control group	30	42.8 ± 1.7	25.0 ± 2.7	0.8 ± 0.1	0.8 ± 0.2
Hypertension during pregnancy	20	43.1 ± 2.6	26.5 ± 2.5	0.9 ± 0.2	1.0 ± 0.1
Pre-eclampsia (mild)	15	44.3 ± 1.6*	30.0 ± 3.3**	1.0 ± 0.2	1.1 ± 0.1
Pre-eclampsia (severe)	10	48.0 ± 2.8**	30.8 ± 3.5**	1.1 ± 0.3	1.2 ± 0.2

Compared with control group *P < 0.05; **P < 0.01.

**Table 2:** Comparison of parameters under 2D conventional echocardiography in four groups of subjects (mean number ± standard deviation).

Projects	Number of cases (pcs)	LAD-ap (mm)	E peak (cm/s)	A-peak (cm/s)	E/A
Control group	30	27.0 ± 2.2	90.0 ± 9.8	70.0 ± 5.0	1.3 ± 0.1
Hypertension during pregnancy	20	28.5 ± 2.1	79.7 ± 5.2*	73.5 ± 5.1	1.1 ± 0.2*
Pre-eclampsia (mild)	15	30.2 ± 2.1**	79.3 ± 4.5*	75.9 ± 4.8*	1.1 ± 0.2*
Pre-eclampsia (severe)	10	33.1 ± 1.4**	77.7 ± 1.6**	78.2 ± 3.3**	0.9 ± 0.1**

Compared with control group *P < 0.05, **P < 0.01.

### Observation of parameters in these four groups of subjects under TDI technique echocardiography

Compared with the control group: E/e was elevated in pregnant women with gestational hypertension compared with the control group, and e was decreased compared with the control group, and the difference was statistically significant (P < 0.05); e was decreased in pregnant women with pre-eclampsia (mild) compared with the control group, and E/e was increased compared with the control group, and the difference was statistically significant (P < 0.05); e was significantly decreased in pregnant women with pre-eclampsia (severe) compared with the control group, and E/e was significantly increased compared with the control group, and the difference was statistically significant (P < 0.01) (Table 3).

**Table 3:** Comparison of parameters under TDI technique echocardiography in four groups of subjects (mean number ± standard deviation).

Projects	Number of cases (pcs)	e (cm/s)	E/e
Control group	30	8.6 ± 0.4	6.5 ± 0.8
Hypertension during pregnancy	20	8.1 ± 0.3*	7.2 ± 0.6*
Pre-eclampsia (mild)	15	8.1 ± 0.3*	7.3 ± 0.7*
Pre-eclampsia (severe)	10	8.0 ± 0.2**	7.5 ± 0.6**

Compared with control group *P < 0.05, **P < 0.01.

### Observation of parameters in these four groups of subjects under 4-dimensional speckle tracking imaging echocardiography

In comparison with the control group, LVESV and LAVI were higher, LVGLS, LVGAS and LAGLS were lower in pregnant women with gestational hypertension compared with the control group, and the difference was statistically significant (P < 0.05). Although LVGRS and LVGCS had slight changes compared with the control group, However, there was no statistical significance (P > 0.05), and LVEDV and LVEF had no significant changes compared with the control group (P > 0.05). LVESV and LAVI in pregnant women with preeclampsia (mild) were significantly higher than those in the control group, LVGLS and LAGLS were significantly lower than those in the control group, the difference was statistically significant (P < 0.01), LVEDV was higher than that in the control group, LVGRS, LVGCS and LVGAS were all lower than those in the control group. The difference was statistically significant (P < 0.05), although LVEF was slightly changed compared with the control group, the difference was not statistically significant (P > 0.05); LVEDV, LVESV and LAVI in preeclampsia (severe) pregnant women were significantly higher than those in the control group, while LVGLS, LVGRS, LVGCS, LVGAS and LAGLS were significantly lower than those in the control group, and the difference was statistically significant (P < 0.01). Although LVEF showed a downward trend, However, the difference was not statistically significant (P > 0.05) (See Tables 4 and 5).

**Table 4:** Comparison of left ventricular parameters under 4-dimensional speckle tracking imaging echocardiography in four groups of subjects (mean number ± standard deviation).

Projects	Number of cases (pcs)	LVEDV (ml)	LVESV (ml)	LAVI (ml/m^2^)	LVEF (%)
Control group	30	104.8 ± 7.3	39.9 ± 5.6	26.1 ± 2.8	59.2 ± 3.2
Hypertension during pregnancy	20	106.8 ± 4.1	43.1 ± 4.1*	27.9 ± 2.3*	58.0 ± 2.1
Pre-eclampsia (mild)	15	109.4 ± 6.8*	44.5 ± 4.6**	28.6 ± 2.8**	57.7 ± 2.2
Pre-eclampsia (severe)	10	111 ± 5.1**	45.3 ± 4.7**	28.9 ± 3.0**	57.6 ± 2.1

Compared with control group*P < 0.05, **P < 0.01.

**Table 5:** Comparison of left ventricular parameters under 4-dimensional speckle tracking imaging echocardiography in four groups of subjects (mean number ± standard deviation).

Projects	Number of cases (pcs)	LVGLS (%)	LVGRS (%)	LVGCS (%)	LVGAS (%)	LAGLS (%)
Control group	30	− 24.5 ± 2.4	25.9 ± 3.1	− 23.5 ± 1.6	24.2 ± 2.7	− 34.8 ± 4.5
Hypertension during pregnancy	20	− 22.8 ± 2.8*	24.4 ± 2.2	− 22.6 ± 1.8	22.5 ± 2.6*	− 31.5 ± 3.9*
Pre-eclampsia (mild)	15	− 18.2 ± 1.7**	23.9 ± 1.9*	− 22.5 ± 1.3*	22.3 ± 2.1*	− 30.1 ± 5.1**
Pre-eclampsia (severe)	10	− 16.3 ± 1.9**	16.1 ± 1.8**	− 15.7 ± 2.0**	19.8 ± 2.8**	− 25.6 ± 2.9**

Compared with control group *P < 0.05, **P < 0.01.

## Discussion

Hypertensive disorders in pregnancy (HDP) are a serious threat to both maternal and fetal health. The rise in prevalence of this disorder and associated health risks have made it one of the most common maternal disorders in recent times^15^. To uncover the underlying pathophysiology, conventional echocardiography has been employed to detect morphological and functional changes in the myocardium^16–18^. Results from recent studies have revealed that LVEDD, LVESD, and LAD-ap were significantly higher in pregnant women with moderate to severe preeclampsia compared to controls. Furthermore, a state of low discharge and higher resistance, leading to an increase in myocardial fiber elongation and left atrial/ventricular internal diameter, was observed in the HDP population. By contrast, patients with gestational hypertension did not demonstrate any statistically significant changes in the inner diameter of the heart chambers, signifying that early HDP does not necessarily result in an increase in the inner diameter of the heart chambers. Left atrial enlargement has been identified as an early sign of HDP^19^. These findings suggest that there is an urgent need for regular, sufficient screening of pregnant women to identify those that may be at risk of developing HDP in order to ensure timely interventions to improve patient outcomes.

The results of this study show that measurements can be made using conventional echocardiography and tissue Doppler imaging (TDI) techniques, suggesting that short-term left ventricular (LV) pressure and volume overload may also lead to diastolic dysfunction^12,20,21^. Furthermore, 4-D speckle tracking (4D-ST) is an attractive technique due to its lack of dependence on heart stereometry, cardiac cycle bias, and single-plane constraints compared to 2-D ST. Moreover, supplementing this non-invasive technique with unique calculation of area strain can help to detect earlier signs of myocardial morphological and functional changes^22^. Our results indicate that LV global longitudinal strain (LVGLS), LV global radial strain (LVGRS), and LV global circumferential strain (LVGCS) were reduced in pregnant women with gestational and pre-eclampsia (mild and severe) compared to a control group, as were LV global area strain (LVGAS) and left atrial global longitudinal strain (LAGLS). Moreover, in both gestational and pre-eclampsia cohorts, a statistically significant reduction was found for LVGLS, LVGRS, LVGCS, LVGAS, and LAGLS. This decrease in strain parameters may be attributed to the decrease of myofilaments in cardiac myocytes, partial loss of myogenic fibronectin, ischemia, and hypoxia in hyperemesis gravidarum, which leads to a relative increase in cardiac stiffness and hence, weakening of myocardial wall motion and smaller amplitude of movements^23,24^. Moreover, in earlier stages of hypertension, namely, gestational hypertension, LVGLS, LVGAS and LAGLS were reduced compared to healthy controls^8,25,26^. The left atrium, an important indicator for cardiovascular prognosis, was also found to be enlarged and had decreased function with lower LAGLS that decreased further for more severe hypertension cohorts. These findings suggest that changes in systolic function and myocardial injury are occurring during the period of gestational hypertension^4,27–29^. 4D-ST has enabled early detection of cardiac dysfunctions in hyperemesis and a more accurate non-invasive assessment of cardiac damage before clinical symptoms manifest. This technique is important for identifying and mitigating further cardiac complications.

In clinical practice, a careful integration and analysis of all parameters of echocardiography should be conducted in patients with hyperemesis gravidarum in order to provide early and accurate assessment of cardiac function. Early and rapid diagnosis is of paramount importance, as it can help clinicians to timely take relevant treatment measures according to the severity of the condition and significantly improve the survival rate of both pregnant women and perinatal infants.

Several limitations to this study need to be acknowledged. Most recent literature data suggest that both the physiological rise in the diaphragm and a narrow antero-posterior chest diameter may contribute to a significant reversible reduction in all the main myocardial strain parameters, assessed by strain echocardiographic imaging in healthy pregnant women. As a consequence the impairment in myocardial strain parameters detected in pregnant women with hypertensive disorders may not be interely ascribed to intrinsic myocardial dysfunction, particularly in those women with anterior chest wall deformity and/or pectus excavatum^30^. In addition, Speckle-tracking analysis suffers from some limitations, such as intervendor variability, dependence on good image quality and temporal stability of tracking pat-terns.

In conclusion, in this study, we reported that TDI combined with 4D myocardial strain parameters can detect early changes in cardiac function of hypertensive disorders in pregnancy, with peak, e, E/A, E/e, LVGLS, LVGAS, and LAGLS being the most sensitive indicators for early changes. Such findings provide a basis for effective clinical treatment of these symptoms.

## Data Availability

The datasets used and/or analyzed during the current study are available from the corresponding author on reasonable request.

## References

[1] Kintiraki E, Papakatsika S, Kotronis G (2015). Pregnancy-induced hypertension. Hormones (Athens).

[2] Yang Z, Yu W (2016). A brief discussion on the Chinese medical understanding and treatment of cardiorenal syndrome. Chin. Tradit. Chin. Med. Acute Care.

[3] Yin X, Xu R, Wang Y (2018). Implication of coronary CT angiography combined with four-dimensional speckle tracking echocardiog- raphy for predicting major adverse cardiac events. Int. J. Cardiovac. Imaging.

[4] Yao J, Xu D, Dai Y (2013). Evaluation of left ventricular systolic pattern and function in normal subjects by area strain. Chin. J. Ultrasound Imaging.

[5] Xie X, Gou WL (2013). Obstetrics and Gynecology.

[6] Lo JO, Mission JF, Caughey AB (2013). Hypertensive disease of pregnancy and maternal mortality. Curr. Opin. Obstet. Gynecol..

[7] Ren L, Yin LX, Wang ZG (2017). Progress in the study of echocardiographic evaluation of left ventricular function in patients with hypertensive disease in pregnancy. Chin. J. Med. Ultrasound.

[8] Nagueh SF, Smiseth OA, Appleton CP (2016). Recommendations for the evaluation of left ventricular diastolic function by echocardiography: An update from the American Society of Echocardiography and the European Association of Cardiovascular Imaging. J. Am. Soc. Echocardiogr..

[9] Yunyou D, Lijun Y, Tiesheng C (2000). Echocardiographic detection of changes in cardiac function during late pregnancy in patients with hyperemesis. Chin. Med. Imaging Technol..

[10] Al-Rikabi ZA, Saeed GT, Haji GF (2016). Estimation of left ventricular diastolic function in gestational hypertension during the third trimester inbaghdad teaching hospital. IOSR J. Dent. Med. Sci..

[11] Aly MF, Kleijn SA, Menken-Negroiu RF (2016). Three-dimensional speckle tracking echocardiography and cardiac magnetic resonance for left ventricular chamber quantification and identifification of myocardial transmural scar. Neth. Heart J..

[12] Yip G, Abraham T, Belohlaviek M (2003). Clinical applications of strain rate imaging. Am. Soc. Echocardiogr..

[13] Gonzalez A, Ravassa S, Lopez B (2018). Myocardial remodeling in hypertension. Hypertension.

[14] Le J, Youji F (2005). Differential Diagnosis of Obstetrical and Gynecological Diseases (fine).

[15] You F, Huo K, Wang R (2012). Maternal mortality in Henan province, China: Changes between 1996 and 2009. PLoS One.

[16] Liu SY, Yang L (2016). Echocardiographic assessment of cardiac morphology and cardiac function changes in late pregnancy in patients with hypertensive disorders of pregnancy. Chin. J. Evid. Based Cardiovasc. Med..

[17] Yue D (2000). Diagnosis and prevention of hyperemesis gravidarum. Chin. J. Pract. Gynecol. Obstet..

[18] Malik R, Kumar V (2017). Hypertension in pregnancy. Adv. Exp. Med. Biol..

[19] Vest AR, Cho LS (2012). Hypertension in pregnancy. Cardiol. Clin..

[20] Singh M, Pathak MS, Paul A (2015). A study on autherogenic indices of pregnancy induced hypertension patients as compare to normal pregnant women. J. Clin. Diagn. Res..

[21] Wei JF (2014). Clinical analysis of 74 cases of combined heart disease in pregnancy. J. Mod. Tradit. Chin. Western Med..

[22] Ponikowski P, Voors AA, Anker SD (2016). 2016 ESC guidelines for the diagnosis and treatment of acute and chronic heart failure. Rev. Esp. Cardiol. (Engl. Ed.).

[23] Li Ya Du, Li MC (2001). Changes in left heart function and atrial structure in patients with hyperemesis. Mod. Adv. Obstet. Gynecol..

[24] Kim MJ, Seo J, Cho KI (2016). Echocardiographic assessment of structural and hemodynamic changes in hypertension-related pregnancy. J. Cardiovasc. Ultrasound.

[25] Sadaniantz A, Kocheril AG, Emaus SP (1992). Cardiovascular changes in pregnancy evaluated by two -dimensional and Doppler echocardiography. J. Am. Soc. Echocardiogr..

[26] Xie MX, Wang XF, Cheng TO (2005). Comparison of accuracy of mitral valve area in mitral stenosis by real -time, threevdimensional echocardiography versus two-dimensional echocardiography versus doppler pressure half-time. Am. J. Cardiol..

[27] Velasco O, Beckett MQ, James AW (2017). Real-time three-dimensional echocardiography: Characterization of cardiac anatomy and function-current clinical applications and literature review update. Biores. Open Access.

[28] Deng BH, Shi WY, Dong NB (2010). Evaluation of real-time three-dimensional echocardiographic measurements of left ventricular volumes in patients with left ventricular remodeling. J. Cardiovasc. Rehabilit. Med..

[29] Zhang QB, Sun JP, Gao RF (2013). Novel single-beat full-volume capture real-time three-dimensional echocardiography and autocontouring algorithm for quantification of left ventricular volume: Validation by cardiac magnetic resonance imaging. Int. J. Cardiol..

[30] Sonaglioni A, Esposito V, Caruso C, Nicolosi GL, Bianchi S, Lombardo M, Gensini GF, Ambrosio G (2021). Chest conformation spuriously influences strain parameters of myocardial contractile function in healthy pregnant women. J. Cardiovasc. Med. (Hagerstown).

